# IL-32**γ** potentiates tumor immunity in melanoma

**DOI:** 10.1172/jci.insight.138772

**Published:** 2020-09-17

**Authors:** Thomas Gruber, Mirela Kremenovic, Hassan Sadozai, Nives Rombini, Lukas Baeriswyl, Fabienne Maibach, Robert L. Modlin, Michel Gilliet, Diego von Werdt, Robert E. Hunger, S. Morteza Seyed Jafari, Giulia Parisi, Gabriel Abril-Rodriguez, Antoni Ribas, Mirjam Schenk

**Affiliations:** 1 Institute of Pathology, Experimental Pathology, and; 2 Graduate School Cellular and Biomedical Sciences, University of Bern, Bern, Switzerland.; 3 Division of Dermatology, Department of Medicine and Department of Microbiology, Immunology and Molecular Genetics, David Geffen School of Medicine at UCLA, Los Angeles, California, USA.; 4 Department of Dermatology, Lausanne University Hospital, Lausanne, Switzerland.; 5 Department of Dermatology, Inselspital, University Hospital of Bern, University of Bern, Bern, Switzerland.; 6 Division of Hematology-Oncology, Department of Medicine, David Geffen School of Medicine at UCLA, and UCLA Jonsson Comprehensive Cancer Center, UCLA, Los Angeles, California, USA.

**Keywords:** Immunology, Oncology, Cancer immunotherapy, Cellular immune response, Melanoma

## Abstract

Myeloid cells orchestrate the antitumor immune response and influence the efficacy of immune checkpoint blockade (ICB) therapies. We and others have previously shown that IL-32 mediates DC differentiation and macrophage activation. Here, we demonstrate that IL-32 expression in human melanoma positively correlates with overall survival, response to ICB, and an immune-inflamed tumor microenvironment (TME) enriched in mature DC, M1 macrophages, and CD8^+^ T cells. Treatment of B16F10 murine melanomas with IL-32 increased the frequencies of activated, tumor-specific CD8^+^ T cells, leading to the induction of systemic tumor immunity. Our mechanistic in vivo studies revealed a potentially novel role of IL-32 in activating intratumoral DC and macrophages to act in concert to prime CD8^+^ T cells and recruit them into the TME through CCL5. Thereby, IL-32 treatment reduced tumor growth and rendered ICB-resistant B16F10 tumors responsive to anti–PD-1 therapy without toxicity. Furthermore, increased baseline IL-32 gene expression was associated with response to nivolumab and pembrolizumab in 2 independent cohorts of patients with melanoma, implying that IL-32 is a predictive biomarker for anti–PD-1 therapy. Collectively, this study suggests IL-32 as a potent adjuvant in immunotherapy to enhance the efficacy of ICB in patients with non–T cell–inflamed TME.

## Abstract 2

IL-32 treatment reprograms DC and macrophages to induce CD8+ T cell infiltration, thereby promoting tumor immunity and response to immunotherapy in murine models of melanoma.

## Introduction

Tumor immunotherapies have emerged as first-line treatment for a number of malignancies (1). In particular, immune checkpoint blockade (ICB) using anti–PD-1 antibodies (nivolumab or pembrolizumab) has demonstrated clinical use in a wide range of cancer types (1, 2). However, the efficacy of ICB is currently limited to a fraction of patients with cancer. The preexisting immune composition of a tumor is a critical determinant of response to ICB, which displays enhanced efficacy in T cell–inflamed tumors (3, 4). In addition to their potential as predictive biomarkers for response to ICB therapy, increased densities of intratumoral CD8^+^ T cells are associated with enhanced overall survival of patients with cancer (5). Currently, there is an unmet need for therapeutic agents that induce T cell infiltration into tumors and permit effective treatment of patients who exhibit resistance or limited response to ICB.

Myeloid cells comprise several subtypes, including DC and macrophages, which play key roles in regulating tumor progression and response to therapies (6). Canonically, mature DC are considered immunogenic, while immature DC are deemed to be immunosuppressive (7). In both mice and humans, *Batf3*-dependent conventional type 1 DC (cDC1) are purported to be the primary DC subpopulation responsible for the cross-priming of tumor-specific CD8^+^ T cells as well as their recruitment to and activation within the tumor (8, 9). Consequently, cDC1 are associated with improved response to ICB in melanoma and increased patient survival in a variety of cancer types (9–11). Similar to DC, macrophages, which are among the most abundant immune cells in tumors, can play bivalent roles in cancer (12). Depending on their phenotype, they are classified as undifferentiated macrophages (M0), classically activated type 1 macrophages (M1), and alternatively activated macrophages (M2) (13). Generally, both M0- and M2-like macrophages have protumorigenic functions, whereas M1-like macrophages are associated with improved survival (14). While DC- and macrophage-targeted agents (e.g., anti-CD40 and poly-ICLC) are being tested in clinical trials, none have demonstrated sufficient efficacy to warrant regulatory approval for solid tumors (15, 16).

In our previous work, we discovered an IL-32–dependent pathway of DC differentiation, and we showed that recombinant IL-32γ (here after referred to as IL-32) induces human DC maturation and activation of CD8^+^ T cells in vitro, suggesting its therapeutic potential in cancer (17). The receptor for IL-32 has not been identified, and no rodent orthologs of IL-32 have been reported (18). Consequently, murine studies with IL-32 have been limited to transgenic mice overexpressing human IL-32 and to the use of recombinant human IL-32 (19–24). However, IL-32 triggers redundant signaling and effector function in human and murine cells (19). IL-32 has pleiotropic functions with 9 known alternative spliced isoforms (18), whereof IL-32γ is the largest and, putatively, the most bioactive isoform (25). Each of these isoforms display different activity, and their respective functions have been studied in a number of diseases, including cancer (22, 26–28). Studies with murine transgenic models showed that overexpression of IL-32 (α, β, and γ) inhibited the growth of murine tumors by inducing tumor cell apoptosis, leading to CD8^+^ T cell responses (21, 22, 29). Nevertheless, transgene-induced intracellular expression of a particular IL-32 isoform may not accurately reflect its mechanistic role in human cancers, given that it may not be secreted. Furthermore, it is difficult to assess the translational relevance of IL-32 treatment in these settings. Hence, we investigated the therapeutic potential of intratumoral IL-32γ administration in murine cancer models, while simultaneously assessing the immunological correlates and prognostic value of high IL-32 expression in human melanoma.

## Results

### IL-32 expression positively correlates with myeloid markers, mature DC, and increased overall survival in patients with melanoma.

**Figure 1:**
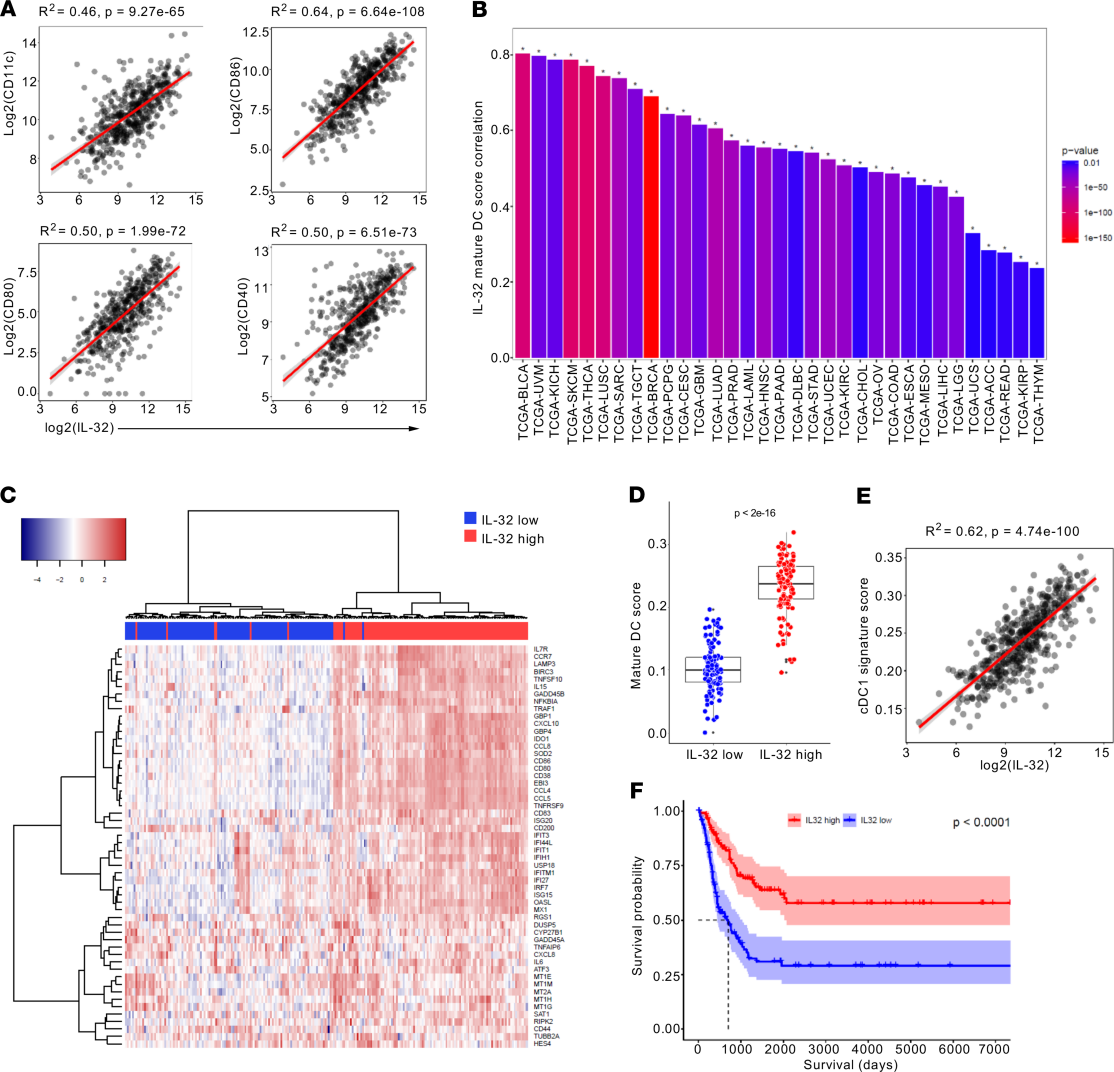
IL-32 expression is associated with activation of myeloid cells and increased overall survival in melanoma. (**A**) Pearson correlation of *IL32* mRNA expression to that of CD11c (*ITGAX*), CD86, CD80, and CD40 in melanoma samples from TCGA. (**B**) Pearson correlation of IL32 gene expression to the mature DC score for all available TCGA cohorts. (**C**) Heatmap of 56 genes defining the mature DC signature, and (**D**) mature DC score in IL-32^lo^– and IL-32^hi^–expressing melanomas (bottom and top 25%, *n* = 118). Differences between groups were analyzed by unpaired, 2-tailed Student’s *t* test. The box extends between 25% and 75%, and the whisker extends up to 75% plus IQR and down to 25% minus IQR. (**E**) Pearson correlation between *IL32* mRNA expression and gene signature score specific for cDC1. (**F**) Kaplan-Meier survival curves for IL-32^lo^ (median survival, 701 days) and IL-32^hi^ (mean survival, not applicable) patients. (**A** and **F**) *n* = 471 biologically independent melanoma samples from TCGA SKCM cohort. (**A**, **D**, and **E**) Each dot represents an individual patient.

In view of the previously demonstrated ability of IL-32 to induce potent DC maturation and macrophage activation, we examined the association between IL-32 and myeloid markers in cutaneous melanoma (17). Therefore, we examined the correlation between IL-32 gene expression and the DC marker CD11c (also known as *ITGAX*) as well as the costimulatory molecules CD40, CD80, and CD86 in melanoma samples from The Cancer Genome Atlas (TCGA). This analysis revealed a significant, positive correlation between IL-32 and genes indicative of activated myeloid cells (Figure 1A). To comprehensively analyze the association between IL-32 expression and mature DC in cancer, we used a previously defined gene signature representing “mature DC” to score samples from all TCGA cohorts (30). This revealed a significant positive correlation of IL-32 gene expression to the mature DC signature not only in melanoma, but in all 33 available TCGA cohorts (Figure 1B). For further analysis, we stratified melanoma samples from TCGA by IL-32 expression to delineate IL-32^hi^ and IL-32^lo^ groups (top and bottom 25%, respectively). Unsupervised clustering analysis confirmed enrichment of genes constituting the mature DC signature in IL-32^hi^ versus IL-32^lo^ melanomas (Figure 1C). Consequently, IL-32^hi^ samples displayed a significantly higher signature score for DC maturation (Figure 1D). Due to the indispensable role of cDC1 in cancer immunity we assessed the relationship between IL-32 and cDC1 in human melanoma. This analysis revealed a significant positive correlation between IL-32 and a previously defined cDC1 gene signature (Figure 1E) (10). These findings provide evidence for a strong association of IL-32 with mature intratumoral DC and increased levels of cDC1. To assess the prognostic relevance of our findings, we performed Kaplan-Meier survival analysis of samples from all TCGA cohorts between IL-32^hi^ and IL-32^lo^ samples (Supplemental Figure 1A; supplemental material available online with this article; https://doi.org/10.1172/jci.insight.138772DS1). The most significant difference in survival between those 2 groups was observed in patients with melanoma, with the IL-32^hi^ group exhibiting markedly increased survival (Figure 1F). Furthermore, a multivariate Cox regression analysis revealed IL-32 as a prognostic parameter after adjustment for other clinical variables (Table 1). Therefore, IL-32 is a potential prognostic biomarker in melanoma; however, this finding requires further validation in a prospective study.

### IL-32^hi^ human melanomas exhibit gene signatures associated with T cell activation, M1 macrophage polarization, and chemokine activity.

**Figure 2:**
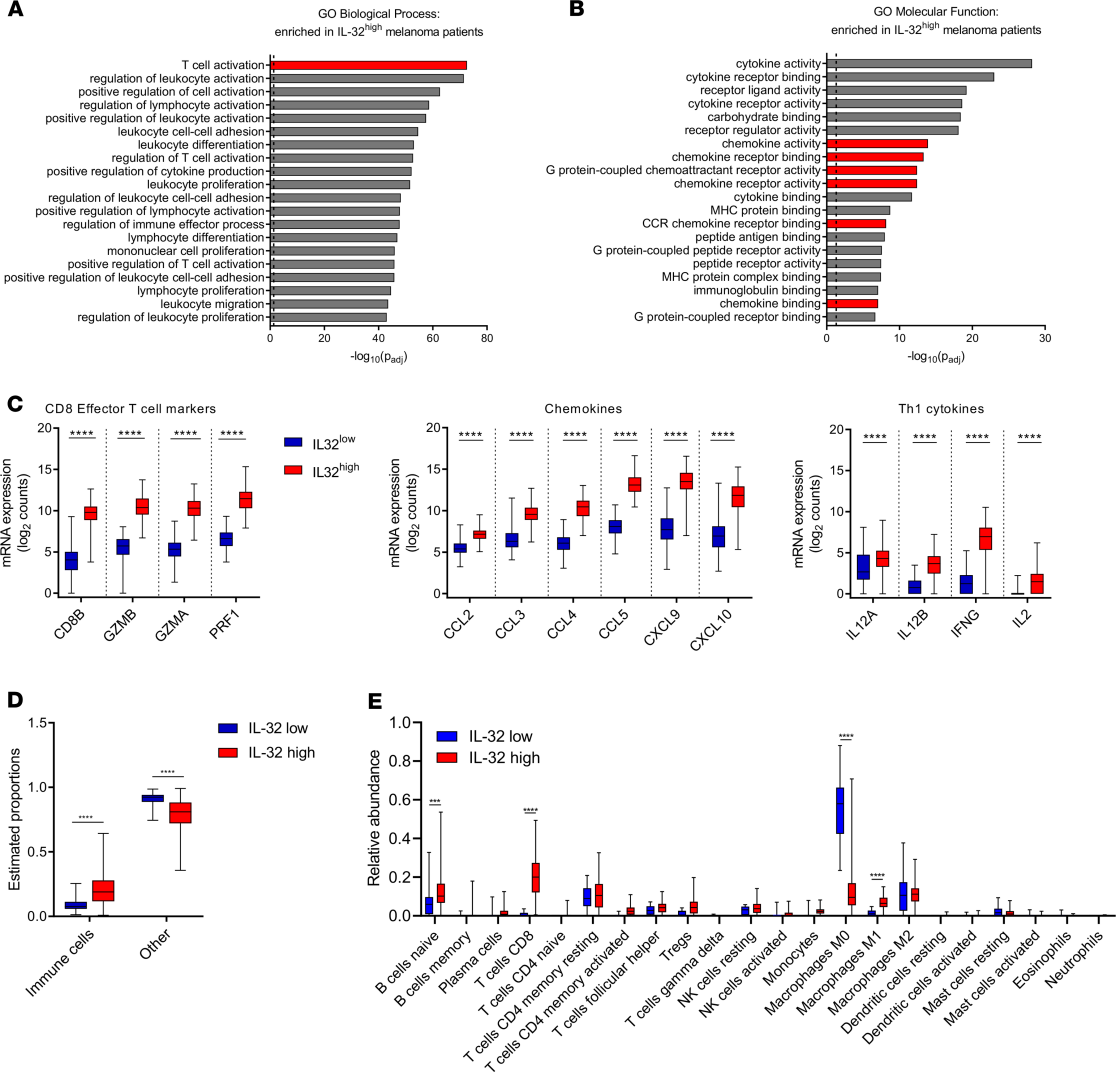
IL-32 expression correlates with a T cell–inflamed tumor microenvironment. (**A** and **B**) Gene ontology term enrichment analysis of genes upregulated in IL-32^hi^ melanomas; shown are the top 20 (**A**) “biological processes” and (**B**) “molecular functions.” Significantly upregulated genes were identified using FDR cutoff of Bonferroni-Hochberg–adjusted *P* = 0.01 and a log_2_ fold change = 1. (**C**) Gene expression of indicated markers for CD8^+^ effector T cells, CD8^+^ T cell–recruiting chemokines and Th1 cytokines in IL-32^lo^ and IL-32^hi^ melanoma samples. Gene expression is shown as normalized, log_2_-transformed counts. (**D**) Proportions of immune cells and nonimmune cells (other) in IL-32^lo^ versus IL-32^hi^ tumors, as estimated by quanTiSeq. (**E**) Relative proportions of indicated immune cell subsets in IL-32^lo^ (*n* = 14) and IL-32^hi^ (*n* = 101) groups, as estimated by CIBERSORT. (**C**–**E**) Data are shown as box-and-whisker plots. The box extends between 25% and 75%, and the whisker extends to the minimum and maximum values. Statistical significance was determined by 2-way ANOVA followed by Šidák’s multiple comparisons test. (**A**–**D**) *n* = 118; ****P < 0.0001.

Next, we dissected the gene expression profiles of the previously defined IL-32^hi^ and IL-32^lo^ melanoma samples. Unsupervised clustering revealed a distinct transcriptional profile in IL-32^hi^ samples compared with that in IL-32^lo^ samples (Supplemental Figure 1B). To delineate biological and molecular functions associated with the IL-32^hi^ gene expression profile in melanoma, we performed gene ontology (GO) term enrichment analysis. Among the most significantly enriched GO terms by classification of “biological processes” and “molecular functions” were those associated with “T cell activation” and “chemokine activity,” respectively (Figure 2, A and B). Consequently, in IL-32^hi^ biopsies, we detected a significantly increased expression of genes associated with CD8^+^ effector T cells as well as chemokines involved in lymphocyte recruitment and Th1-associated cytokines (Figure 2C) (31, 32). These findings suggest that IL-32^hi^ melanomas exhibit a highly chemotactic tumor microenvironment (TME) favoring T cell infiltration into the tumor. Therefore, we studied the relationship between IL-32 gene expression and tumor-infiltrating immune cells using quanTIseq, a recently described computational approach to estimate the relative proportions of various tumor infiltrating immune cells from bulk tumor RNA-Seq profiles (33). This analysis revealed significantly higher proportions of immune cells in IL-32^hi^ tumors and fewer nonimmune cells (stroma and tumor), relative to IL-32^lo^ melanomas (Figure 2D). To further dissect the relative proportions of immune cell subpopulations, we used CIBERSORT (cell-type identification by estimating relative subsets of RNA transcripts) (34). Importantly, IL-32^hi^ tumors displayed increased proportions of CD8^+^ T cells and M1 macrophages but reduced M0 (unpolarized) macrophages, relative to IL-32^lo^ samples (Figure 2E). DC, which represent a minor fraction of immune cells in melanoma, were largely undetectable by CIBERSORT. In addition, we detected increased frequencies of tumor-infiltrating lymphocyte (TIL) in the pathology slides associated with TCGA cutaneous melanoma samples from IL-32^hi^ tumors (Supplemental Figure 1C). Collectively, these findings suggest that IL-32 activates DC and induces M1 macrophage polarization, leading to the induction of a chemotactic, T cell–inflamed TME.

### IL-32 promotes myeloid cell activation and chemokine activity.

**Figure 3:**
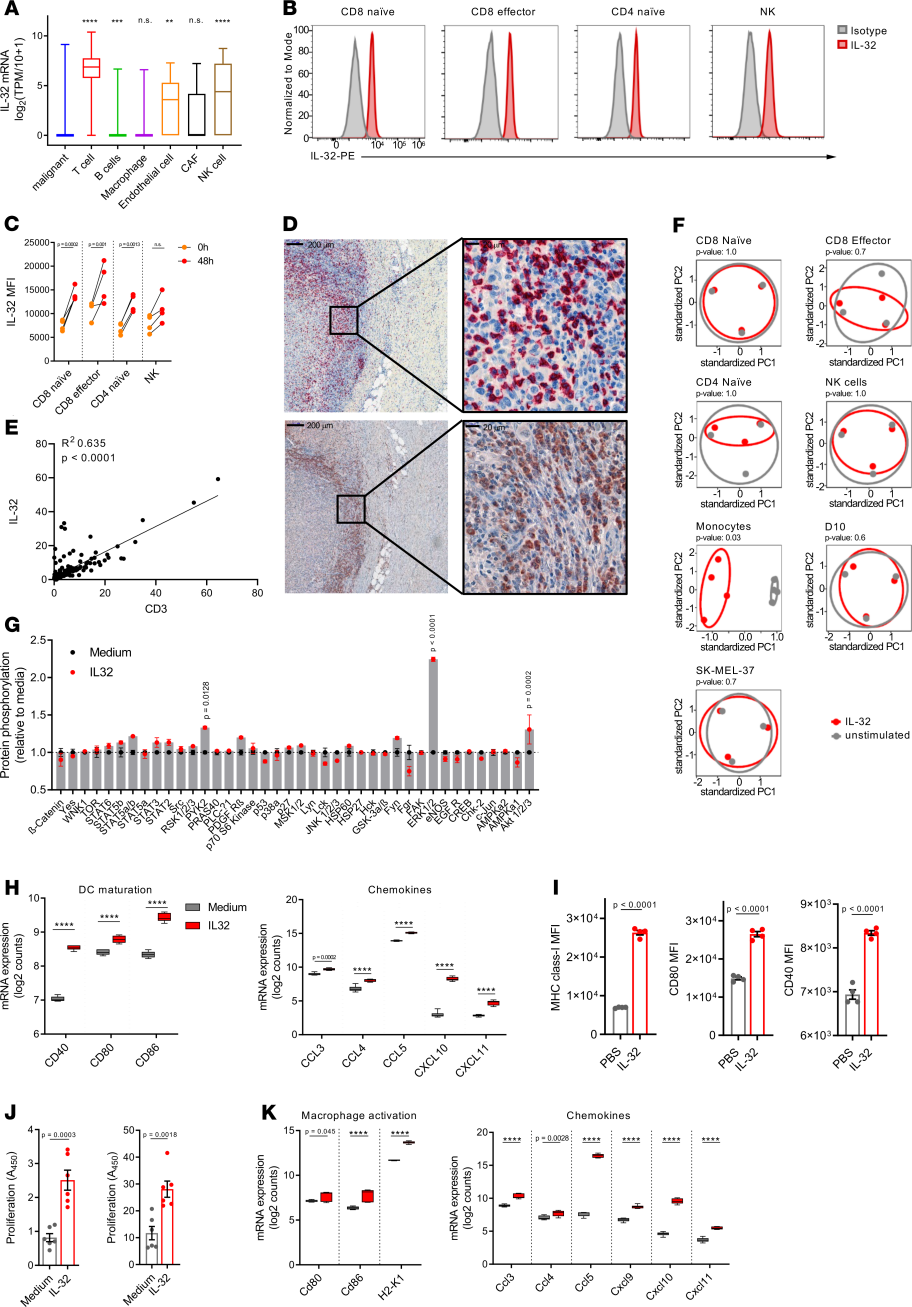
Lymphocyte-derived IL-32 mediates activation of myeloid but not lymphoid or melanoma cells. (**A**) IL-32 expression of indicated cell types determined by single-cell RNA-Seq data obtained from GSE72056. Data are shown as box-and-whisker plots. The box extends between 25% and 75%, and the whiskers extend to the minimum and maximum. Statistical significance was determined using 1-way ANOVA followed by Tukey’s multiple comparisons test. (**B**) Representative histograms of IL-32 expression in indicated immune cell populations from healthy human blood assessed by flow cytometry (*n* = 4). (**C**) IL-32 mean fluorescence intensity (MFI) in each immune cell subset treated for 48 hours with anti-CD3/CD28 and IL-2 or left untreated, (*n* = 4). (**D**) Representative immunohistochemical labeling for CD8^+^ T cells (top) and IL-32 (bottom) in serial sections (*n* = 3). Original magnification, ×5; ×40 (high-magnification images). Scale bars: 200 μm; 20 μm (high-magnification images). (**E**) Correlation between IL-32^+^ and CD3^+^ cells, as assessed by immunofluorescence labeling of a melanoma TMA, indicated as a percentage of DAPI^+^ cells (*n* = 154; from 140 individual patients) (**F**) PCA from NanoString-derived gene expression profiles of purified human PBMC subsets and melanoma cell lines treated with IL-32 for 24 hours or left untreated (monocytes, *n* = 4; other PBMC subsets, melanoma cell lines, *n* = 3). Statistical significance was determined using Adonis in the vegan package in R. (**G**) Kinase phosphorylation levels measured by phospho-kinase array in monocytes treated with IL-32 for 20 minutes or left untreated. Two biologically independent samples were measured in duplicates. (**H**) RNA expression levels of the indicated DC maturation markers and chemokines in IL-32–treated (24 hours) and untreated murine BMDC (*n* = 6). (**I**) MFI of the indicated DC maturation markers assessed by flow cytometry on IL-32–treated versus untreated murine BMDC (*n* = 4), assessed after 48 hours. (**J**) Proliferation of OT-I cells cocultured with IL-32–treated or untreated BMDC after 48 hours measured using a BrdU cell proliferation assay kit (BioVision). BMDC were either pulsed with OVA protein (left) or SIINFEKL peptide (right) (*n* = 6). (**I** and **J**) Data shown as mean ± SEM; unpaired, 2-tailed Student’s *t* test. (**K**) Gene expression levels of the indicated macrophage activation markers and T cell–attracting chemokines in murine BMDM treated for 24 hours with IL-32 or left untreated, measured by NanoString (*n* = 6). (**H** and **K**) Normalized, log_2_-transformed mRNA counts shown as box-and-whisker plots. The box extends between 25% and 75%, and the whiskers extend to the minimum and maximum. (**C**, **G**, **H**, and **K**) Statistical significance was determined using 2-way ANOVA followed by Šidák’s multiple comparisons test. **P < 0.01, ***P < 0.001, ****P < 0.0001.

To assess the cellular source of IL-32 within the tumor, we examined previously annotated single-cell RNA-Seq data from patients with melanoma (35). IL-32 expression was highest in T lymphocytes and significantly higher in NK and endothelial cells compared with cancer cells (Figure 3A). Furthermore, we combined microarray gene expression data sets of 9 human hematopoietic cell populations and 63 melanoma cell lines, which confirmed that IL-32 is predominantly expressed in certain lymphocyte populations (CD8^+^ and CD4^+^ T cells and NK cells) compared with other immune cell subsets or melanoma cell lines (Supplemental Figure 2A) (36, 37). Next, we measured IL-32 protein expression in peripheral blood lymphocytes from healthy human donors using flow cytometry (Figure 3B). Following activation of human PBMC in vitro with anti-CD3/CD28 and IL-2, intracellular protein expression of IL-32 significantly enhanced in naive and effector CD8^+^ and naive CD4^+^ T cells (Figure 3C). These findings were further validated by immunohistochemical labeling of formalin-fixed, paraffin-embedded serial sections of human melanoma tissue. We observed IL-32 protein in lymphocyte-rich regions in close apposition with CD8^+^ T cells (Figure 3D). A more comprehensive analysis using multiplex immunofluorescence showed a substantial overlay of IL-32 with CD3 and CD8 (Supplemental Figure 2B) and a correlation between the percentage of IL-32^+^ and CD3^+^ T cells (Figure 3E). Furthermore, IL-32 expression was significantly higher in a group of long-term survivors as compared with that in a group of short-term survivors (Supplemental Figure 2C). In order to examine the effects of IL-32 on the expression of over 500 immune-related genes in both human melanoma and immune cell subsets, we used the NanoString Human Immunology Panel V2. We treated established human melanoma cell lines (D10, SK-Mel-37) and multiple FACS-purified immune cell subsets from healthy donor PBMC with IL-32. PCA revealed an altered gene expression profile upon IL-32 treatment in monocytes but not in lymphocytes or melanoma cell lines (Figure 3F). To assess the direct downstream signaling of IL-32 on human monocytes, we performed a phospho-kinase array analysis. Phosphorylation was most strongly induced on ERK-1/2 (Figure 3G), which was also observed in murine cells (data not shown), indicating that IL-32 specifically binds to a receptor, resulting in the downstream activation of the MAPK/ERK pathway in myeloid cells. Furthermore, we detected no direct cytotoxicity of IL-32 to human and mouse melanoma cells (Supplemental Figure 3A). Ingenuity Pathway Analysis (IPA) revealed the enrichment of multiple identical pathways in both human monocytes and murine BMDC treated with IL-32 (Supplemental Figure 3, B and C). Furthermore, IL-32–matured BMDC displayed significantly elevated mRNA levels of DC maturation markers and T cell–recruiting chemokines (Figure 3H), similar to the gene signature associated with IL-32^hi^ human melanomas. Flow cytometric analysis of IL-32–treated BMDC confirmed an increased surface expression of MHC class I and costimulatory molecules (CD40, CD80) (Figure 3I). Accordingly, IL-32–matured BMDC displayed enhanced cross-presentation of exogenous OVA antigen as well as increased MHC-I presentation of peptide antigen (SIINFEKL) to prime OT-I CD8^+^ T cells (Figure 3J). These data are consistent with our previous data showing enhanced CD8^+^ T cell activation in human IL-32–derived DC in vitro (17). Given the increase in M1 macrophage frequencies concomitant with reduction of unpolarized M0 macrophages in IL-32^hi^ tumors (Figure. 2E), we also addressed the response of bone marrow–derived macrophages (BMDM) to IL-32 using NanoString gene expression profiling. IL-32 treatment significantly increased the expression of macrophage activation markers and T cell–recruiting chemokines, especially Ccl5 (Figure 3K). Compared with BMDC, IL-32–induced Ccl5 expression was more pronounced in BMDM. Taken together, these results suggest that IL-32 is primarily expressed by lymphocytes and mediates its effect by triggering the MAPK/ERK pathway in myeloid cells, thereby inducing their maturation, activation, and chemokine secretion.

### IL-32–treated tumors exhibit increased lymphocyte infiltration and activation of CD8^+^ effector T cells.

**Figure 4:**
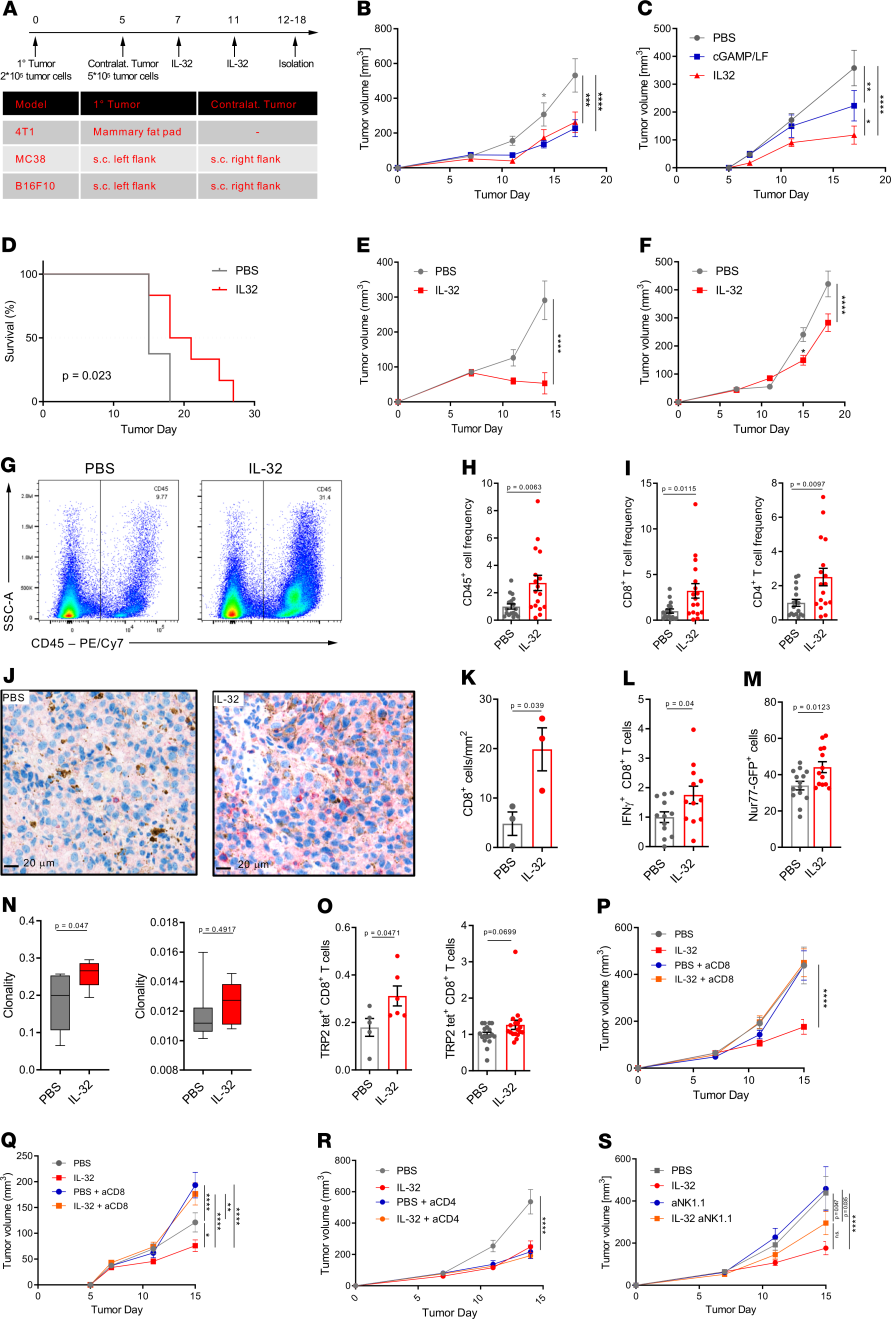
IL-32 induces a systemic CD8^+^ T cell–mediated tumor-specific immune response. (**A**) Experimental setup for in vivo tumor treatments. MC38 and B16F10 were inoculated in C57BL/6J mice, and 4T1 tumors in BALB/c mice (**A**–**O**). (**B**) Growth curves of IL-32–, cGAMP-, or PBS-treated primary and (**C**) contralateral, nontreated B16F10 melanomas. *P < 0.05, **P < 0.01, ***P < 0.001. Data are representative of 4 independent experiments, with *n* = 6 mice per group. (**D**) Kaplan-Meier survival curves of B16F10-bearing mice treated with IL-32 (*n* = 6) or PBS (*n* = 8). (**E**) Representative growth curves of IL-32–treated and untreated MC38 colon adenocarcinoma (*n* = 6) and (**F**) orthotopic 4T1 breast tumors (*n* = 10). (**G–O**) On day 14, the primary treated tumors and spleens were harvested for flow cytometric analyses, IHC or TCRβ chain sequencing. (**G**) Representative flow cytometry plots displaying frequencies of CD45^+^ immune cells for each treatment group and (**H**) their quantification shown as relative frequencies (*n* = 18). (**I**) Relative frequencies of CD8^+^ and CD4^+^ T cells as a percentage of live cells (*n* = 18). (**J**) Representative immunohistochemical staining and (**K**) morphometric enumeration as cells/mm^2^ of CD8^+^ T cells (*n* = 3). Scale bar: 20 μm. (**L**) Relative frequencies of IFN-γ^+^ cells as percentage of CD8^+^ T cells (*n* = 12). (**M**) Frequencies of Nur77-GFP^+^ cells of CD8^+^ T cells, as determined by flow cytometry in B16F10-inoculated and PBS- (*n* = 14) and IL-32–treat (*n* = 13) Nur77 mice. (**N**) TCR clonality in tumors (left, PBS, *n* = 7; IL-32, *n* = 5) and spleens (right, PBS, *n* = 7; IL-32, *n* = 6), representative of 2 independent experiments. (**O**) Relative frequencies of TRP-2 tetramer–positive cells, as a proportion of CD8^+^ T cells in tumors (left, *n* = 6) and spleens (right, *n* = 18). (**P**) Growth curves of IL-32–treated and untreated B16F10 primary tumors (PBS, IL-32, PBS + aCD8, *n* = 18; IL-32 + aCD8, *n* = 16) and (**Q**) untreated contralateral tumors with or without CD8 depletion (*n* = 16–18). *P = 0.0171; **P = 0.003; ****P < 0.0001. (**R**) Growth curves of IL-32–treated and untreated B16F10 primary tumors with or without CD4 depletion (*n* = 11) or (**S**) NK depletion (PBS, *n* = 12; IL-32, *n* = 18). (**B**, **C**, **E**, **F**, and **P**–**S**) Growth curves are shown as mean ± SEM, using 2-way ANOVA followed by Šidák’s multiple comparisons test. (**D**) Comparison of survival curves was performed using a log-rank (Mantel-Cox) test. (**H**, I, **K**–**M**, and **O**) Data are displayed as mean ± SEM. Statistical significance was determined by unpaired, 2-tailed Student’s *t* test. ****P < 0.0001.

Given our findings showing a beneficial role of IL-32 in human melanoma, we investigated the efficacy of IL-32 as a tumor immunotherapy in mice. As such, we established a standard treatment regimen to evaluate the efficacy of IL-32 in ICB-resistant B16F10 and 4T1 as well as in highly immunogenic MC38 murine syngeneic tumor models (Figure 4A) (38–43). Activation of STING by cGAMP can induce systemic tumor immunity and was used as a positive control (44). In the B16F10 model, intratumoral administration of IL-32 as well as cGAMP significantly reduced the tumor growth in the treated primary tumors (Figure 4B) as well as in the untreated contralateral tumors (Figure 4C). However, IL-32 treatment was more effective in the contralateral tumors, demonstrating its ability to induce potent systemic tumor immunity. Intravenous IL-32 administration showed a similar effect on tumor growth (Supplemental Figure 5A). Mice treated with IL-32 showed a 30% prolonged survival compared with PBS, with a median survival of 19.5 and 15 days, respectively (Figure 4D). We confirmed the therapeutic efficacy of IL-32 in subcutaneously injected MC38 colon adenocarcinomas (Figure 4E) and orthotopically inoculated 4T1 mammary carcinomas (Figure 4F). Flow cytometric profiling revealed a nearly 3-fold increase in CD45^+^ leukocytes (Figure 4, G and H) as well as CD8^+^ and CD4^+^ T cell frequencies in IL-32–treated B16F10 tumors (Figure 4I). A similar increase of CD8^+^ and CD4^+^ T cells in the TME was observed if IL-32 was administered systemically (Supplemental Figure 5, B and C). To confirm intratumoral T cell recruitment, we performed CD8 immunoperoxidase staining on tissue sections of B16F10 tumors. IL-32–treated tumors displayed an approximately 3-fold increase in absolute numbers of CD8^+^ T cells within the tumor (Figure 4, J and K) as well as significantly increased frequencies of IFN-γ^+^ (Figure 4L) and Nur77-GFP^+^ CD8^+^ T cells (Figure 4M). Taken together, these findings denote significantly enhanced CD8^+^ T cell activation following IL-32 treatment. Furthermore, TCR clonality was significantly enhanced in the tumors, but not in the spleens, of mice treated with IL-32 (Figure 4N), suggesting the presence of tumor-reactive T cell clones. In order to determine whether IL-32 treatment resulted in increased tumor antigen-reactive CD8^+^ T cells, we assessed their specificity for MHC class I tetrameric complexes bearing SVYDFFVWL peptides derived from tyrosinase-related protein 2 (TRP-2). TRP-2 is a melanocyte-specific antigen in human melanoma with a known ortholog in murine B16F10 cells, and CD8^+^ TIL specific for TRP-2 have been identified in both humans and mice (45). Indeed, the frequencies of TRP-2-tetramer–positive CD8^+^ T cells were significantly increased in tumors, but not in the spleens, of IL-32–treated mice (Figure 4O). To assess whether an increase in the frequency of tumor-reactive CD8^+^ T cells is essential for IL-32–mediated control of tumor growth, we performed antibody-based depletion of CD8^+^ T cells in mice concurrently receiving IL-32. CD8^+^ T cell depletion abolished the efficacy of IL-32 in both the primary treated tumor and untreated contralateral tumor (Figure 4, P and Q). In contrast, IL-32 treatment efficacy was largely independent of CD4 or NK cells, as assessed by CD4 (Figure 4R) and NK depletion (Figure 4S), respectively. Notably, depletion of CD4 T cells is known to reduce the growth of B16 tumors (46, 47). Collectively, these data show that an increased infiltration and activation of CD8^+^ T cells is indispensable for IL-32–induced tumor control.

### IL-32 induces immune cell recruitment and limits tumor growth via the induction of a chemokine-rich TME.

**Figure 5:**
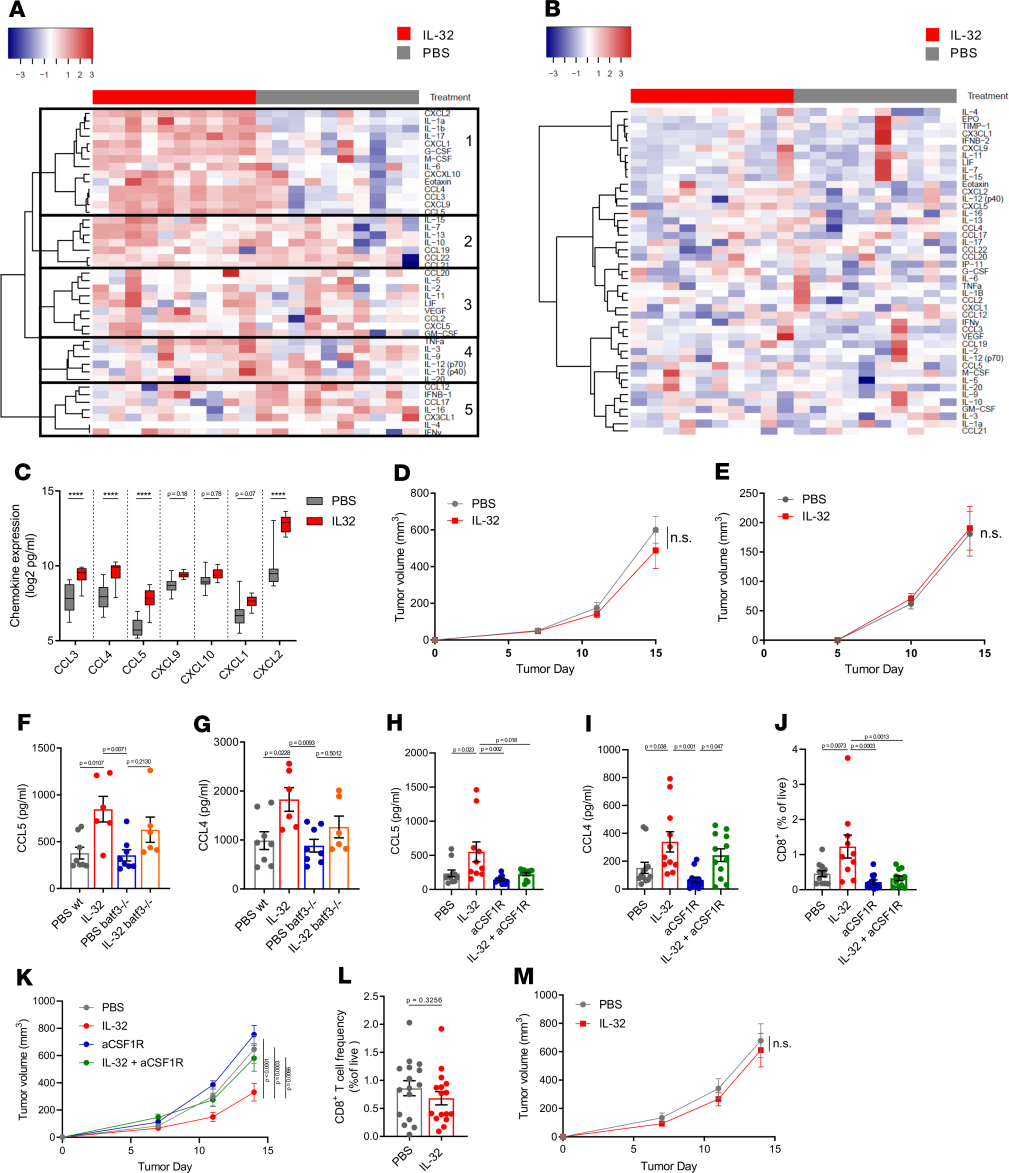
IL-32 treatment efficacy relies on the generation a proinflammatory, chemokine-rich TME. (**A**–**I**) Mice bearing B16F10 tumors were treated with IL-32 or PBS as in Figure 4A. (**A**–**C**) At day 12, tumors and spleens were isolated and lysed. Cytokine and chemokine levels were assessed using multiplexed bead array (*n* = 10, from 2 independent experiments). Data are represented as log_2_(pg/mL). (**A**) Hierarchical clustering of cytokine protein levels in tumor lysates and (**B**) sera. (**C**) Protein expression levels of the indicated cytokines and chemokines from tumor lysates, shown as box-and-whisker plot; the box extends between 25% and 75%, and the whiskers extend to the minimum and maximum values. (**D**) Growth curves of IL-32– or PBS-treated primary (*P* = 0.2824) and (**E**) contralateral (*P* = 0.9859) B16F10 tumors inoculated in *Batf3^–/–^* mice. (**F**) CCL5 and (**G**) CCL4 expression levels in B16F10 tumors at day 12 after tumor inoculation in B6 WT and *Batf3*^–/–^ mice, as determined by ELISA (PBS, *n* = 8; IL-32, *n* = 6). (**H–K**) IL-32– or PBS-treated B16F10 tumors in mice with or without macrophage depletion using anti-CSF1R mAb. (**H**) CCL5 (PBS, aCSF1R, IL-32 + aCSF1R, *n* = 11; IL-32, *n* = 10) and (**I**) CCL4 (PBS, aCSF1R, IL-32 + aCSF1R, *n* = 12; IL-32, *n* = 11) expression levels in B16F10 tumors at day 14 after tumor inoculation determined by ELISA. (**J**) Corresponding CD8^+^ T cell infiltration, as determined by flow cytometry (PBS, aCSF1R, IL-32 + aCSF1R, *n* = 12; IL-32, *n* = 10) and (**K**) tumor growth curves (PBS, aCSF1R, IL-32 + aCSF1R, *n* = 6; IL-32, *n* = 5). (**L** and **M**) *CCR5^–/–^* mice (*n* = 16) were inoculated with B16F10 cells. (**L**) At day 14, tumors were isolated and CD8^+^ T cell frequencies were determined by FACS. Statistical analysis was performed using 2-tailed Student’s *t* test. (**M**) Growth curves of IL-32– or PBS-treated B16F10 tumors in *CCR5^–/–^* mice (*P* = 0.9325). (**D**, **E**, **K**, and **M**) Tumor growth curves are depicted as mean ± SEM. Differences between groups were determined using 2-way ANOVA followed by Šidák’s multiple comparisons test. ****P < 0.0001.

To obtain a more comprehensive analysis of the changes induced by IL-32 within the TME and systemically, we analyzed primary B16F10 tumor homogenates and sera from IL-32– and PBS-treated mice for the expression of 44 key cytokines and chemokines using an addressable laser bead immunoassay (ALBIA). Using hierarchical clustering analysis followed by application of a threshold to linkage distance, we delineated 5 protein clusters in tumor samples, of which cluster 1 contained co-upregulated chemokines and proinflammatory cytokines in IL-32–treated tumors (Figure 5A). In contrast, there was no difference observed in the sera, indicating no induction of systemic inflammation markers, such as IL-6 and TNF-α (Figure 5B). Further dissection of cluster 1 induced in IL-32–treated tumors showed significantly increased protein levels of the CCR5-binding chemokines CCL3, CCL4, CCL5, and CXCL2 (Figure 5C), corroborating the observations in IL-32^hi^ human melanoma. To assess the functional relevance of cross-presenting cDC1 in IL-32–induced tumor control, we examined the effects of IL-32 treatment in B16F10 tumors inoculated in *Batf3*^–/–^ mice according to the established treatment regimen (see Figure 4A).

The capacity of IL-32 to control tumor growth was abrogated in *Batf3^–/–^* mice, both in the primary (Figure 5D) and untreated contralateral tumors (Figure 5E). In addition, IL-32–induced CD8^+^ T cell infiltration was abrogated in *Batf3^–/–^* mice (Supplemental Figure 4). However, the differences in CCL5 and CCL4 expression between IL-32–treated tumors from WT and *Batf3^–/–^* mice were not statistically significant (Figure 5, F and G), suggesting that DC are not exclusively responsible for the increased tumor chemokine levels in response to IL-32 treatment. In contrast, macrophage depletion using anti-CSF1R antibodies completely abrogated IL-32–induced CCL5 but not CCL4 expression (Figure 5, H and I). Together with the results from Figure 3K, our data suggest that macrophages within the TME secrete CCL5 in response to IL-32. Accordingly, the CD8^+^ T cell infiltration (Figure 5J) and tumor growth reduction (Figure 5K) was lost in the absence of macrophages. Since the expression of CCR5 ligands was significantly associated with IL-32 gene expression in human melanoma tumors and induced following IL-32 treatment, we investigated the effects of IL-32 in *CCR5*^–/–^ mice. In the absence of CCR5, IL-32 treatment did not result in increased frequencies of CD8^+^ T cells (Figure 5L) and displayed no effects on tumor growth (Figure 5M). These results indicate that IL-32 efficacy is predicated on inducing TIL recruitment and thus modulating the immune phenotype of the TME.

### IL-32 potentiates the effects of ICB in mice and is predictive for response to anti–PD-1 therapy in patients with melanoma.

**Figure 6:**
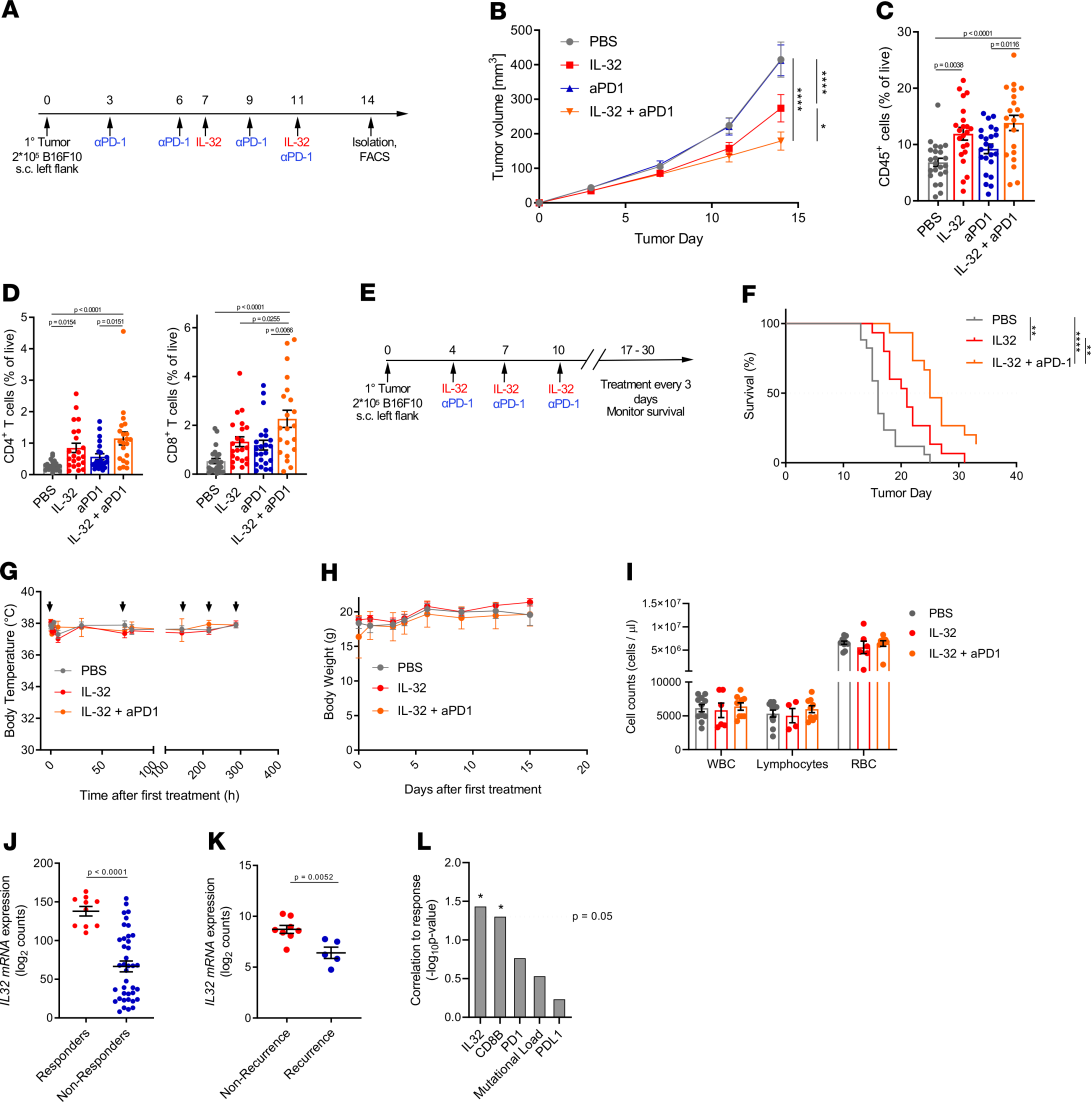
IL-32 treatment is synergistic with concurrent anti-PD1 in mice, and IL-32 expression is predictive for response to anti-PD1 therapy in patients with melanoma. (**A**) Experimental setup for B16F10 dual treatment with IL-32 and anti–PD-1 antibody used in **B–D**. (**B**) Tumor growth shown as mean ± SEM (IL-32, aPD1, IL-32 + aPD1, *n* = 23; PBS, *n* = 24). Statistical significance was determined by 2-way ANOVA followed by Šidák’s multiple comparisons test. *P = 0.0199, ****P < 0.0001. (**C**) Frequencies of CD45^+^ immune cells and (**D**) CD4^+^ T cells and CD8^+^ T cells as a proportion of viable cells (*n* = 21–23). *P* values were computed by 1-way ANOVA followed by Tukey’s multiple comparisons test. Data are represented as mean ± SEM. (**E**) Experimental setup for survival and safety assessment with additional IL-32 and anti–PD-1 treatments used for **F**–**J**. (**F**) Kaplan-Meier survival curves. Significance was determined by log-rank test (IL-32, IL-32 + aPD-1, *n* = 15; PBS, *n* = 17). (**G**) Body temperature and (**H**) body weight of mice upon treatment (*n* = 6). (**I**) White blood cell (WBC), lymphocyte, and red blood cell (RBC) counts as cells/μl blood (*n* ≥ 4). Blood was obtained when mice were euthanized. Differences between groups were determined using 2-way ANOVA followed by Šidák’s multiple comparisons test. (**J**) *IL32* mRNA expression levels in biopsies from patients with melanoma before anti–PD-1 (nivolumab) treatment. (**K**) *IL32* mRNA expression in biopsies of patients with melanoma receiving neoadjuvant pembrolizumab treatment (nonrecurrence, *n* = 8; recurrence, *n* = 5). The data set was obtained from GSE123728. (**J** and **K**) *P* values were computed by 2-tailed, unpaired Student’s *t* test. Error bars show mean ± SEM. (**L**) Multivariate logistic regression between response to nivolumab and mRNA expression of the indicated genes or mutational load. (**J** and **L**) Patients were stratified into responders (complete response and partial response, *n* = 10) and nonresponders (stable disease or progressive disease, *n* = 39). The data set was obtained from GSE91061.

The increased density of TIL is generally associated with improved responses to ICB, in particular to anti–PD-1 and anti-PDL1 antibodies (48). Due to our results showing that IL-32 enhanced intratumoral T cell infiltration, we investigated the capacity of IL-32 to induce responsiveness in non-ICB–responding melanomas. Therefore, we established a dual treatment regimen using IL-32 and anti–PD-1 in B16F10 tumors (Figure 6A). Mice receiving both IL-32 and anti–PD-1 showed significantly reduced tumor growth compared with mice receiving either monotherapy (Figure 6B) and displayed the most significant increase in CD45^+^ leukocytes (Figure 6C). Similarly, the frequencies of both CD4^+^ and CD8^+^ T cells were most significantly enhanced in the group treated with a combination of IL-32 and anti–PD-1 (Figure 6D). To assess the safety and long-term effects of IL-32 as single-agent treatment and in combination with anti–PD-1 therapy, we established a new treatment schedule (Figure 6E). The survival rate was significantly improved in mice receiving a combination of IL-32 and anti–PD-1 (median survival, 25 days), which is about a 19% increase compared with IL-32 alone (median survival, 21 days), and 56% compared with PBS (median survival, 16 days) (Figure 6F). IL-32 treatment with or without anti–PD-1 did not result in a significant change of body temperature (Figure 6G) or body weight (Figure 6H). Furthermore, white blood cell (WBC), lymphocyte, and red blood cell (RBC) counts (Figure 6I) remained constant upon treatment with IL-32 alone or in combination with anti–PD-1. Together with the serum cytokine profiling (Figure 5B), these data suggest IL-32 as a safe tumor treatment and suggest that it is also safe when combined with anti–PD-1 therapy. Subsequently, we analyzed IL-32 gene expression in biopsies from patients with melanoma before treatment with anti–PD-1 (nivolumab or pembrolizumab) (49, 50). *IL32* mRNA levels were significantly higher in anti–PD-1 treatment–responding patients with melanoma or patients with no disease recurrence compared with nonresponders or patients with recurrent disease in 2 independent cohorts (Figure 6, J and K). Several predictive biomarkers for ICB have been identified, including the tumor mutational load, CD8 infiltration, and PD-1 and PD-L1 expression (1). However, none of these biomarkers are truly predictive individually (1, 48), demonstrating the need for additional biomarkers. Here, we compared the predictive potential of *IL-32* to *PD-1*, *PD-L1*, *CD8B*, and the mutational load in pretreatment samples from the Nivolumab cohort (49). Intriguingly, IL-32 showed the most significant correlation to response, while CD8B expression also reached significance (Figure 6L). Therefore, baseline intratumoral IL-32 levels represent a potential predictive biomarker for response to ICB; however, this needs further validation. Together, our findings suggest that IL-32 in combination with anti–PD-1 is a viable and save strategy for inducing tumor immunity in a wide range of immune excluded tumor types, which fail to respond to ICB monotherapy (3).

**Figure 7:**
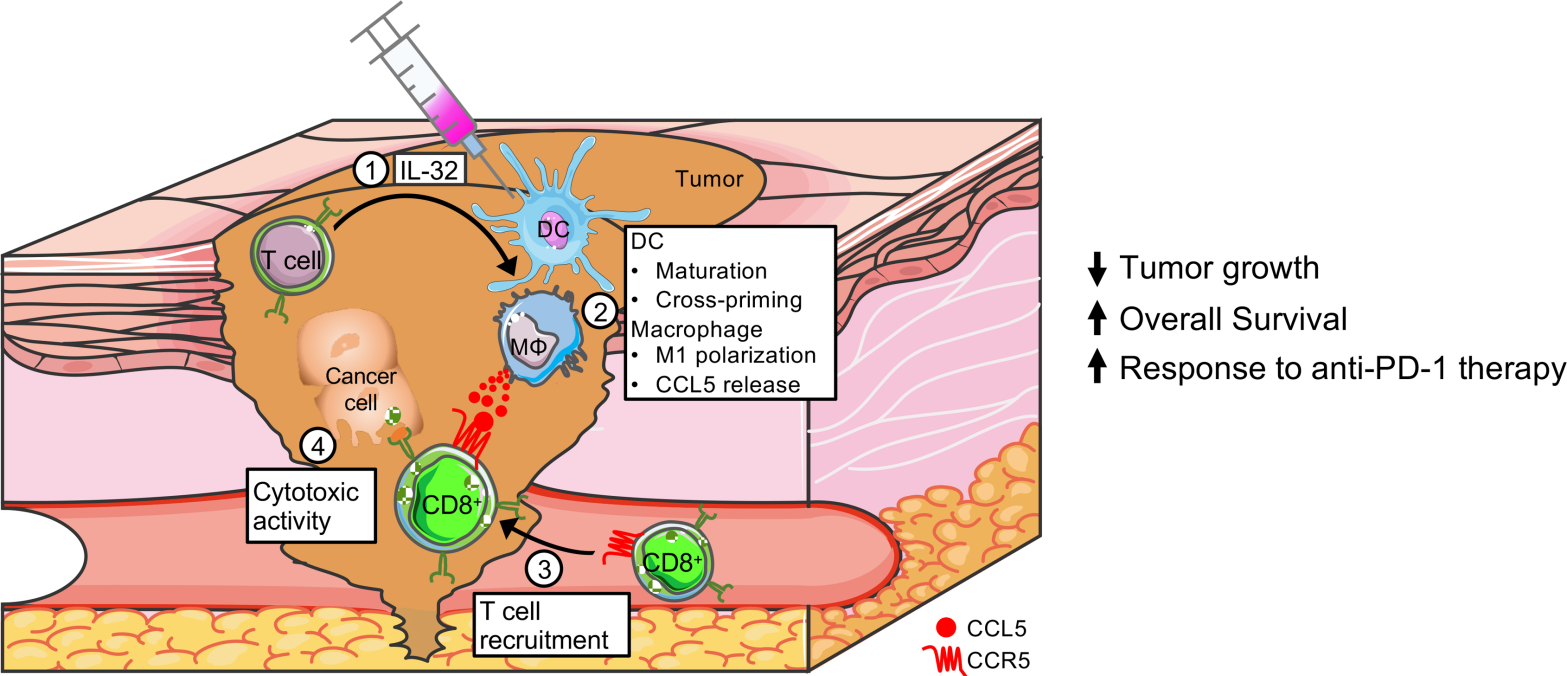
Roles of IL-32 in the antitumor immune response. In melanoma, IL-32 is mainly produced by T cells. Injections of IL-32 improve DC function and trigger M1 polarization as well as CCL5 release in macrophages, resulting in CCR5-mediated CD8^+^ T cell infiltration into the TME and the eradication of cancer cells. Accordingly, IL-32 treatment has antitumorigenic functions in murine cancer models and positively correlates with overall survival of patients with melanoma. Moreover, it acts synergistically with anti–PD-1 therapy and strongly correlates with response to anti–PD-1 therapy in patients with melanoma.

Overall, we revealed a detailed mechanism of action of IL-32 in melanoma (Figure 7). First, we found that IL-32 is primarily expressed in tumor-infiltrating T cells and acts in a paracrine fashion on myeloid cells. Specifically, it induced maturation and cross-priming in DC as well as M1 polarization and CCL5 release in macrophages. Hence, CD8^+^ T cell infiltration into the TME via CCR5 was enhanced, leading to the killing of cancer cells. Consequently, IL-32 injections reduced the growth of various syngeneic tumors, and IL-32 expression was associated with prolonged overall survival of patients with melanoma. Moreover, given the ability of IL-32 to induce a T cell–inflamed TME, it potentiated the response to anti–PD-1 therapy and strongly correlated with response to pembrolizumab and nivolumab in patients with melanoma.

## Discussion

A T cell–inflamed TME is generally associated with better prognosis as well as improved response to ICB (1, 5, 48). In this study, we demonstrate that IL-32 potentiates T cell infiltration into the tumor, leading to enhanced survival of patients with melanoma and response to ICB. Injection of IL-32 in murine tumors induced a systemic, tumor-specific immune response. Furthermore, IL-32 treatment rendered B16F10 tumors responsive to anti–PD-1 treatment and the combination significantly improved control of tumor growth compared with either monotherapy. Our results indicate that T cell–derived IL-32 acts on DC to induce maturation and cross-presentation function, as well as on macrophages to trigger M1 polarization and CCL5 expression. Thus, in response to IL-32, DC and macrophages act in concert to prime and recruit T cells into the TME. Collectively, these findings provide robust evidence for the therapeutic efficacy of IL-32 as a tumor immunotherapy, in particular, for patients whose cancers exhibit immune excluded TME.

Mature DC and M1-polarized macrophages regulate the antitumor immune response and are positive prognostic factors for patients with various cancers (14, 51–53). We show that IL-32 gene expression positively correlates with mature DC in all 33 examined cancer types from TCGA. In melanoma, increased IL-32 expression was also associated with higher levels of cDC1 and improved overall survival. Notably, *Batf3*-dependent cDC1 are the primary DC population orchestrating the antitumor immune response within the TME (8). Deficiency of cross-presenting DC (in *Batf3^–/–^* mice) or CD8^+^ T cells abrogated the therapeutic efficacy of IL-32 in mice, suggesting that the IL-32 effector mechanisms are dependent on cDC1 priming of tumor-specific CD8^+^ T cells. Additionally, we found a positive correlation between IL-32 and M1 macrophages concomitant with a negative correlation to M0 macrophages in melanoma, suggesting that IL-32 induces both cross-presentation in DC and M1 polarization in macrophages.

While IL-32 is associated with the outcome of several cancers and plays a role in both inflammatory and infectious diseases, its precise cellular source and functions within the TME remain poorly described (18). A recent report demonstrated an association between high IL-32 expression and a dedifferentiated phenotype in cell lines, but not in samples from patients with cutaneous melanoma (54). This observed discrepancy between IL-32 expression in cell lines versus whole tumors may be attributed to the source of IL-32 in human cancers. Our study strongly suggests the primary cellular source of IL-32 in human tumors to be T cells. This may explain the previously noted positive correlation between IL-32 expression and individual genes indicative of lymphocyte infiltration, such as *CD3E*, *CD8A*, and *IFNG*, in bulk tumor samples (54). This observation is further corroborated by the initial study describing NK4 (later termed IL-32) as a T cell– and NK cell–specific transcript, which was not detected in HeLa carcinoma or HL60 and K562 myeloid leukemia cell lines (55).

Our results also provide potentially new mechanistic insight into the role of IL-32, demonstrating that myeloid cells but not lymphocytes or cancer cells respond to IL-32 treatment by activating the MAPK/ERK pathway, suggesting its binding to a hitherto unidentified surface receptor. Studies addressing the role of IL-32 in mice are limited by the fact that no murine homolog has been identified so far (18). However, our data derived from gene expression profiling of IL-32–treated human monocytes and murine BMDC indicate redundant functions of IL-32 in human and mice. In addition to its redundant effector functions, we discovered identical downstream phosphorylation patterns upon IL-32 activation of murine DC as observed in humans. Together, our data suggests that in human melanoma IL-32 is primarily expressed in lymphocytes and enhances the activation of intratumoral myeloid cells in a paracrine fashion. As such, IL-32 represents a promising modulator of intratumoral DC and macrophage function and thus, a potentially novel molecular target for myeloid cell–based cancer immunotherapy. Notably, as opposed to studies performed in IL-32–overexpressing mice, we did not observe any direct cytotoxicity of IL-32 to tumor cells in vitro (21, 22). This might be due to distinct effects of intracellular IL-32 overexpression versus those of exogenously added IL-32γ.

CD8^+^ T cell recruitment to the tumor is governed by multiple chemokines. In melanoma, CD8^+^ T cell infiltration correlates with the expression of several chemokines, including the CCR5 ligands, CCL4 and CCL5, as well as the CXCR3 ligands, CXCL9 and CXCL10 (31). Recent evidence suggests that CCL4 and CCL5, but not CXCL9-11, are positively associated with survival in melanoma (56). Our transcriptomic analyses of IL-32^hi^ melanoma samples revealed an enhanced T cell infiltration, likely as a result of the increased levels of the aforementioned T cell–recruiting chemokines. In line with the human data, the most salient feature of IL-32–treated B16F10 melanomas was a chemokine-rich TME, concomitant with enhanced frequencies of activated, tumor-specific CD8^+^ T cells, recapitulating the findings from human melanoma TCGA samples. Since IL-32 treatment mainly induced CCR5 ligands, we inoculated *Ccr5*-deficient (*Ccr5*^–/–^) mice with B16F10 melanomas. *Ccr5* deficiency abrogated the IL-32–mediated CD8^+^ T cell infiltration and its therapeutic effect, i.e., tumor growth reduction, highlighting the induction of T cell–recruiting chemokines as a key effector mechanism of IL-32 in tumor immunity. Our data are supported by earlier in vitro observations that IL-32–treated murine BMDC upregulate a number of chemokines, in particular CCL5, and can induce migration of activated CD4^+^ and CD8^+^ T cells in vitro (24). Notably, we found that the induction of chemokine expression in response to IL-32 was mainly dependent on macrophages, as macrophage depletion completely abrogated the induction of CCL5 upon IL-32 treatment.

A preexisting tumor-specific T cell response is a critical determinant of the patient survival and response to ICB (1, 5). However, the TME is often highly immunosuppressive, with several mechanisms preventing infiltration and activation of tumor-specific CD8^+^ T cells (57). As such, ICB is currently being tested in combination with a number of additional TME-modulating therapies, including proinflammatory cytokines (58). Here, we show for the first time to our knowledge that direct administration of IL-32 leads to increased frequencies of tumor-infiltrating T cells in multiple poorly immunogenic mouse tumor models. Thus, IL-32 in combination with ICB may be a viable therapeutic strategy for ICB-resistant patients with non–T cell–inflamed tumors (4). Our data provide proof of concept for this approach, with no detectable immune-related toxicity in these animals. In addition, our data suggest that IL-32 gene expression could delineate patients with melanoma who respond to nivolumab or pembrolizumab, highlighting its potential use as a predictive biomarker for response to anti–PD-1 therapy. While additional studies are required to validate IL-32 as a predictive or prognostic biomarker and to translate IL-32 as a tumor immunotherapy to the clinic, the therapeutic efficacy of this cytokine for melanoma treatment has been demonstrated in this study.

## Methods

### Tissue culture.

Murine B16F10 melanoma and 4T1 breast carcinoma cell lines were purchased from ATCC. The MC38 colon adenocarcinoma cell line was provided by A. Zippelius (Department of Biomedicine, University of Basel, Basel, Switzerland). Human SK-MEL-37 and D10 melanoma cell lines were a gift from P. Zajac (Department of Biomedicine, University of Basel). All murine cell lines were cultured in complete RPMI-1640 medium (MilliporeSigma; supplemented with 10% FBS, 100 units/mL penicillin, 100 μg/mL streptomycin, 1 mM sodium pyruvate, and 2 mM L-glutamine). Human melanoma cell lines were grown in complete DMEM (MilliporeSigma; supplemented with 10% FBS, 100 units/mL penicillin, 100 μg/mL streptomycin, 1 mM sodium pyruvate, and 2 mM L-glutamine). Primary murine cells, such as splenocytes and T lymphocytes and human PBMC, were cultured using complete RPMI-1640 medium as described above.

### BMDC maturation and T cell proliferation assays and BMDM activation.

To generate murine BMDC or BMDM, bone marrow was flushed from femurs and tibias of C57BL/6J (B6) mice and cultured in Petri dishes at 5 × 10^6^ cells per dish using complete RPMI-1640, supplemented as described above and containing 10 ng/mL GM-CSF (MilliporeSigma) or M-CSF (culture medium supplemented with 20% L929 supernatant), respectively. GM-CSF or M-CSF were replaced on day 4. After 1 week, cells were harvested and BMDC were further purified using magnetic anti-CD11c microbeads (Miltenyi Biotec). Purified CD11c^+^ murine DC or BMDM were seeded into 6-well plates and matured with 200 ng/mL human IL-32 (R&D Systems) for 48 hours. DC were pulsed with 1 mg/mL OVA (EndoFit Ovalbumin, InvivoGen) or 1 nM SIINFEKL (MilliporeSigma) antigen for 24 hours. Subsequently, CD8^+^ T cells were purified from the spleens of OT-I mice using EasySep Mouse CD8^+^ T cell Isolation Kit (negative selection, STEMCELL Technologies) and cocultured with matured CD11c^+^ DC. T cell proliferation was assessed after 48 hours using the BrdU Cell Proliferation Assay Kit (BioVision). Purified IL-32γ matured CD11c^+^ DC and BMDM as well as untreated controls were also transcriptionally profiled after 24 hours using NanoString and analyzed via flow cytometry after 48 hours.

### Monocyte isolation and differentiation.

For human monocyte mRNA profiling via NanoString, blood was obtained from healthy volunteers (Interregionale Blutspende SRK). PBMC were isolated using Ficoll (GE Healthcare) and enriched for monocytes using the EasySep Human Monocyte Enrichment Kit without CD16 Depletion (STEMCELL Technologies). Purified monocytes were treated with 200 ng/mL human IL-32 (R&D Systems) for 24 hours or left untreated.

### Mice, tumor inoculation, and in vivo studies.

B6 and BALB/cJ mice were purchased from Janvier Labs (France). *Batf3*-deficient (*Batf3*^–/–^) mice on a B6 background, generated as previously described (59), were obtained from M. Suter (Department of Research, Bavarian Nordic GmbH, Martinsried, Germany; University of Zurich, Zurich, Switzerland). OT-I–transgenic mice (TgTcraTcrb)1100Mjb (60), were obtained from University of Zürich (Animal Management System – iRATS). *CCR5^–/–^* mice on a B6 background were obtained from The Jackson Laboratory. *Nur77-GFP* mice (Tg(Nr4a1-EGFP/cre)820Khog/J) were obtained from S. Freigang (Institute of Pathology, University of Bern). Eight to ten-week-old age- and sex-matched animals were used for all experiments. B16F10 melanoma and MC38 colon cancer cells were engrafted s.c. (2 × 10^5^ cells) onto the left flank of B6 mice on day 0. For certain experiments, a second contralateral tumor was induced s.c. in (5 × 10^5^ cells) on day 5. Mice were inoculated with breast cancer tumors by injecting 4T1 cells (2 × 10^5^ cells) into the mammary fat pads of BALB/c mice. After randomization, mice were treated with intratumoral or intravenous (where indicated) injection of recombinant human IL-32γ (5 μg/mouse) or PBS into the primary tumor on day 7 and 11 while leaving the contralateral tumor untreated. For all tumor models, mice were euthanized between day 12 and 18 after tumor inoculation (see Figure 4A) or when tumor volume exceeded 1000 mm^3^. Anti–PD-1 (RMP1-14, Bio X Cell, BE0146) mAb was administered i.p. (200 μg) biweekly starting at day 3 after tumor induction. For depletion of specific lymphocyte subsets, mice were treated i.p. with anti-CD8 antibody (200 μg, clone 53-6.7, Bio X Cell, BE0004-1), anti-CD4 antibody (200 μg, clone GK1.5, Bio X Cell, BE0003-1), or anti-NK1.1 antibody (200 μg, clone PK136, Bio X Cell, BE0036) every other day starting from day 3 after tumor induction. Macrophages were depleted by administering an anti-CSF1R antibody (500 μg, clone AFS98, Bio X Cell, BE0213) every other day starting 5 days before tumor inoculation. Tumor size was measured in 2 dimensions using a digital caliper in a blinded fashion Tumor volume was calculated using the following formula *V* = (length × width^2^)/2 (44). For survival experiments, mice were euthanized when the tumor volume reached 1000 mm^3^ or when ulcerations occurred. WBC and RBC counts were determined using a Sysmex KX-21N Automated Hematology Analyzer. All mice were housed in specific–pathogen free conditions in the Central Animal Facility of the University of Bern.

### Flow cytometric analyses and cell sorting.

To obtain single-cell suspensions for flow cytometry, tumors were mechanically dissociated and filtered twice through a 40 μM strainer (Thermo Fisher Scientific). The following antibodies against mouse antigens were used for flow cytometry: anti–IFN-γ (XMG1.2, BioLegend, 505830), anti-CD3ε (145-2C11, BioLegend, 100306), anti-CD3 (17A2, BioLegend, 100236), anti-CD4 (RM4-5, BioLegend, 100538), anti-CD8α (53-6.7, BioLegend, 100730), anti-CD45.2 (104, BioLegend, 109830), anti-CD40 (3/23, BioLegend, 124624), anti-CD80 (16-10A1, BioLegend, 104708), anti-H-2K^b^ (MHC class I, AF6-88.5, BioLegend, 116518), anti-CD11c (N418, BioLegend, 117336), and anti-CD86 (GL-1, BioLegend, 105006). For human antigens, the following antibodies were used: anti-CD45RO (UCHL1, BioLegend, 304224), anti-CD45RA (JS-83, Thermo Fisher Scientific, 12-9979-42), anti-CD197 (G043H7, BioLegend, 353226), anti-CD62L (DREG-56, Thermo Fisher Scientific, 47-0629-42), anti-CD3 (OKT3, BioLegend, 317328), anti-CD4 (RPA-T4, BioLegend, 300518), anti-CD8 (SK1, BioLegend, 344722), anti-IL32αβγδ (KU32-52, BioLegend, 513503), and anti-CD56 (REA196, BioLegend, 304611). The Zombie Aqua or UV Fixable Viability Kit (BioLegend, 423102 or 423108) was used to distinguish between live and dead cells in each experiment. PE-streptavidin (Thermo Fisher Scientific, SA10041) was used to detect biotinylated anti-IL32, whereas all other antibodies were directly conjugated to fluorochromes. All the reagents mentioned above were purchased from BioLegend or Thermo Fisher Scientific. Prior to surface staining of cells, Fc receptor blocking was performed with anti-mouse CD16/32 (2.4G2, generated in-house) for 15 minutes. Subsequently, cell surface markers were stained with antibodies in FACS buffer (PBS with 2% FBS and 1 mM EDTA) for 45 minutes on ice. Intracellular staining for IL-32 and IFN-γ was performed using the eBioscience Foxp3/Transcription Factor Staining Buffer Set following the manufacturer’s protocols. Trp2 tetramers (iTAg Tetramer/PE H-2K^b^ TRP2) were obtained from MBL and added to the surface stain antibody cocktail. Samples were analyzed on BD LSRII and Beckman Coulter CytoFLEX S flow cytometers and data were processed using Flowjo (Tree Star). Naive and effector CD4^+^ and CD8^+^ T cells were FACS purified using a Moflo Astrios EQ cell sorter (Beckman Coulter). Naive cells were defined as CD45RA^+^CD45RO^–^CCR7^+^CD62L^+^ and effector cells were identified as CD45RA^+^CD45RO^–^CCR7^–^CD62L^–^ as previously defined (61).

### Tumor cytokine bead array and chemokine ELISA.

B16F10 melanomas were established and treated as described (see Figure 4A). On day 12, mice were sacrificed, and tumors and peripheral blood samples (cardiac puncture) were collected. Tumors were homogenized in a lysis buffer (4 μl/mg) consisting of PBS and 0.05% Tween-20 as well as leupeptin (100 μM), aprotinin (10 μg/mL), and PMSF (200 μM). Lysis was performed with the aid of the QIAGEN TissueLyser II platform. Tumor lysates were centrifuged at 10,000*g* for 10 minutes at 4°C, and supernatants were collected, aliquoted, and stored in –80°C until analysis. Sera were obtained from blood by centrifugation in BD Microtainer blood collection tubes (BD SST). Protein concentrations of tumor lysates were measured using Bradford protein quantification, and normalized samples and sera were submitted to Eve Technologies for cytokine array analysis (Mouse Cytokine Array/Chemokine Array 44-plex) or measured using a CCL5 or CCL4 specific ELISA (Abcam). Cytokine protein concentration log_2_(pg/mL) were visualized using the heatmap3 package in R (62).

### DNA isolation and TCRβ sequencing.

On day 14 after tumor inoculation, B16F10 tumors and spleens were isolated. Genomic DNA was extracted using the DNeasy Blood and Tissue Kit (QIAGEN). TCRβ sequencing was performed using the ImmunoSEQ survey level assay (Adaptive Biotechnologies). Sequencing data was analyzed using the ImmunoSEQ analyzer (Adaptive Biotechnologies).

### TCGA data collection and analysis.

TCGA gene expression data as well as clinical information from all available cancer cohorts were obtained using GDCRNATools (63). Counts were normalized using default parameters of the DESeq2 package in R (64). Gene expression of IL-32 was correlated with gene expression of CD11c (*ITGAX*), *CD86*, *CD80*, and *CD40* respectively, using Pearson correlation. For further analyses, samples were stratified in 2 groups according to their expression of IL-32 (low and high) using the bottom and top quartiles. The mature DC gene signature was obtained from a list of significantly upregulated genes induced in LPS plus IFN-γ matured human DC as described in a previous study (30). Bulk tumor RNA-Seq data sets from all TCGA cohorts were scored for the mature DC and cDC1 (*CLEC9A, XCR1, CLNK, BATF3*) signatures using the singscore package in R (65). For cutaneous melanoma (SKCM), enrichment of genes constituting the mature DC signature were visualized using the heatmap3 package in R (62). The association between *IL32* mRNA expression and survival was assessed by Kaplan-Meier analysis using the survminer package in R (http://www.sthda.com/english/rpkgs/survminer/). GO term enrichment analyses were performed using the clusterProfiler package in R (66). For the Kaplan-Meier survival plot in Figure 1F, the survival time was calculated by subtracting the days from initial diagnosis to diagnosis of the tumor used for RNA-Seq (“days_to_submitted_specimen_dx” taken from http://firebrowse.org/?cohort=SKCM, file “Merge_Clinical (MD5)“) from the overall survival time. The relative abundance of immune and nonimmune cells was estimated from bulk tumor gene expression profiles from TCGA using quanTIseq (33). Furthermore, the relative proportions of 22 types of infiltrating immune cell subsets were determined via the LM22 leukocyte signature matrix using CIBERSORT (34). A recommended *P* value threshold of less than 0.05 on the *P* value for the global deconvolution of each sample was applied (67).

### Analysis of GEO data sets.

Publicly available microarray data sets were obtained from gene expression omnibus (GEO). Single-cell RNA-Seq data from CD45^+^ and CD45^–^ FACS-purified cells from human melanoma samples was obtained from GSE72056 (35). Affymetrix arrays were batch normalized and processed using the fRMA package in R (68). Samples with a generalized normalized unscaled standard error (GNUSE) ≥1.25 were excluded. RNA-Seq data from patients with nivolumab treatment were obtained from GSE91061 and normalized using the DESeq2 package in R (49, 64). NanoString samples from patient biopsies with pembrolizumab treatment were obtained from GSE123728 and normalized with nSolver analysis software 4.0 (NanoString) (50).

### NanoString mRNA profiling.

FACS-purified human PBMC subsets from healthy human donors were lysed in 10 μl Buffer RLT (QIAGEN) containing 1% 2-mercaptoethanol (MilliporeSigma) diluted 1:2 in PBS. Subsequently, 5 μl lysate was directly used for mRNA profiling. Human melanoma cells as well as murine BMDC and BMDM were lysed in 250 μL RLT buffer as described above and purified using RNeasy Mini Spin Columns (QIAGEN) according the manufacturer’s protocol. Prior to measurement, the RNA concentration of all samples was normalized. Human cell lysates were assessed for the expression of key immunological genes using NanoString Human Immunology Panel V2. Mouse BMDC lysates were transcriptionally analyzed using the Mouse Immunology Panel. All profiling was performed using the nCounter Digital Analyzer (NanoString Technologies). Data quality control, normalization, and evaluation were performed using the nSolver analysis software 4.0.

### Immunohistochemistry and immunofluorescence.

All samples were obtained from patients with resected primary or metastatic melanoma. Serial sections were cut from formalin-fixed, paraffin-embedded tissue blocks. Murine B16F10 tumors were established and treated as in Figure 4A and embedded in paraffin on day 14. For CD8^+^ T cell detection, human melanoma and B16F10 tissue sections were processed for deparaffinization, target retrieval, and immunohistochemical labeling using the in-house established fully automated Bond RX system (Leica Biosystems). Human CD8 antigen was detected using a mouse anti-human CD8 antibody (C8/144) while mouse CD8 antigen was detected using a rat polyclonal anti-mouse CD8 antibody (Dianova) and alkaline phosphatase. For IL-32 detection, human melanoma tissue sections were deparaffinized using Neo-Clear (Merck Millipore) and graded ethanol washing, followed by antigen retrieval (DAKO Target Retrieval Solution pH6) in an autoclave (Prestige Medical). IL-32 was detected using a mouse anti-human IL-32 antibody (KU32-09), EnVision+ System- HRP Labelled Polymer anti-mouse (Dako), and an AEC Staining Kit (MilliporeSigma). Counterstaining was performed with hematoxylin dye. Whole-slide images were acquired using a Pannoramic 250 Flash II (3D Histech). Immunofluorescence labeling for IL-32 (KU32-52, BioLegend), CD3 (SP7, Abcam), CD8 (C8/144B, Biosystems), and DAPI was performed using the Opal 4-Color Manual IHC kit (Akoya Biosciences) according to the manufacturer’s protocol. Cell quantification was performed with the assistance of the image analysis software QuPath (69).

### Phospho-kinase array.

Human monocytes were isolated as described above using the EasySep Human Monocyte Enrichment Kit without CD16 Depletion (STEMCELL Technologies). Monocytes were treated with IL-32γ (200 ng/mL) and incubated at 37°C for 20 minutes. Sample preparation and array procedure was performed according to the manufacturer’s protocol using the Proteome Profiler Phospho-Kinase Array (R&D Systems). Signal measurement was performed using the ChemiDoc Touch Imaging System (Bio-Rad) and quantified using the Protein Array Analyzer tool in ImageJ (NIH).

### Statistics.

Statistical analyses were performed using GraphPad Prism 7.0 or R. Statistical significance was determined as described in the figure legends, with 2-way ANOVA followed by Šidák’s multiple comparisons test and 2-tailed Student’s *t* test. R^2^ was calculated as follows: (Pearson correlation coefficient)^2^. All measurements were taken from distinct samples; *n* = biological independent replicates. *P* < 0.05 was considered significant.

### Study approval.

Patient samples used in this study were collected from patients with metastatic melanoma. Human melanoma tissue was collected in accordance with guidelines of the Cantonal Ethics Committee (KEK) in Bern under approved protocols (KEK ID: 2017-02246). All animal experiments were performed in accordance with federal regulations and approved by the Cantonal Veterinary Office.

## Author contributions

TG and MS contributed to overall project design. TG, MS, and HS wrote the manuscript. TG, MS, HS, MK, NR, LB, DVW, and GP performed the in vitro and in vivo animal experiments, which were analyzed by TG and HS. FM conducted the collection of human melanoma tissue sections used for immunohistochemical staining. REH provided human melanoma tissue sections and SMSJ provided the TMA. TG performed computational analyses of TCGA and GEO data sets and prepared all figures. MG, RLM, AR, and GAR provided intellectual input.

## Supplementary Material

Supplemental data

## Figures and Tables

**Table 1:**
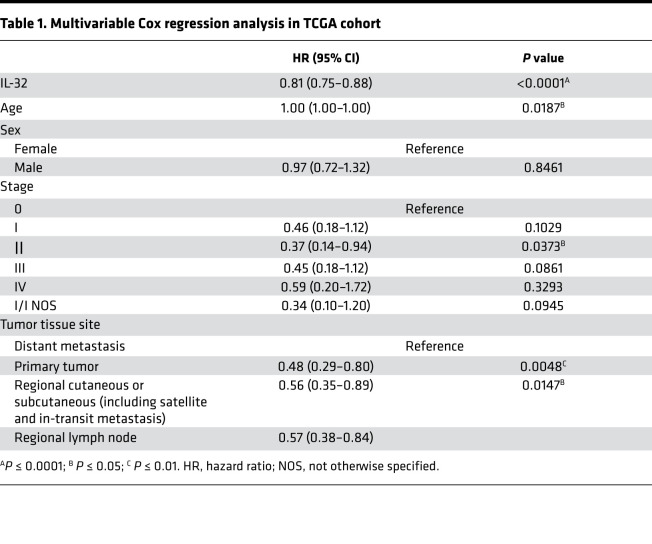
Multivariable Cox regression analysis in TCGA cohort

## Version 1 08/25/2020

In-Press Preview

## Version 2 09/17/2020

Electronic publication
