# Supplementary material for: IL-32γ potentiates tumor immunity in melanoma
Source: JCI Insight. 2020 Sep 17;5(18):e138772. doi: 10.1172/jci.insight.138772 (PMC7526542; doi:10.1172/jci.insight.138772)
Supplement: Supplemental data [file jciinsight-5-138772-s250.pdf]

Supplemental Material:

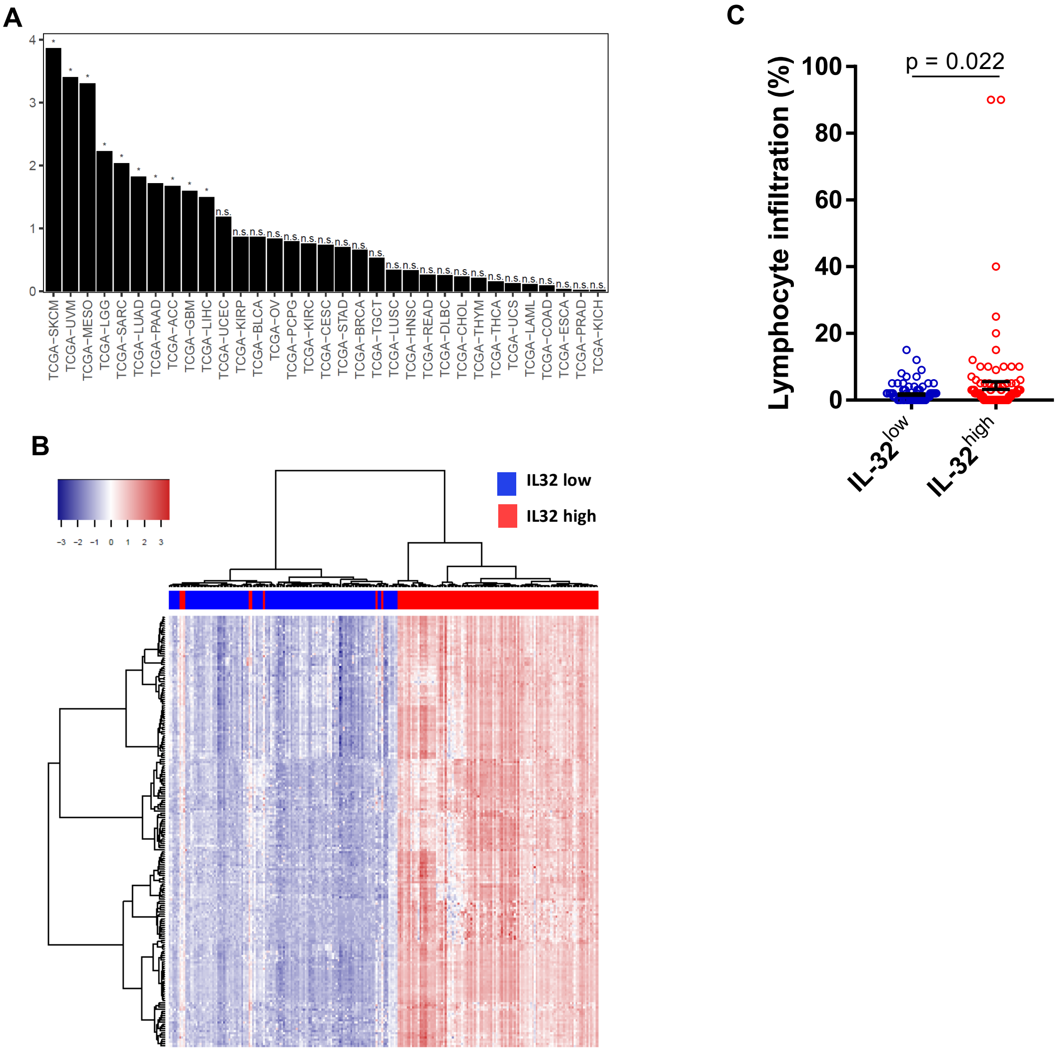

Supplemental Figure 1. Comparison of overall survival and infiltration of mature DC and T cells between IL-32^{high} and IL-32^{low} cancer patients from TCGA. (**A**) P-values are derived from Kaplan-Meier survival analysis comparing IL-32^{low} vs. IL-32^{high} patients (bottom and top 25%,) in all TCGA cohorts. *p-value < 0.05; non-significant (n.s.). (**B**) Heatmap and hierarchical dendogram depicting the top 200 differentially expressed genes between IL-32^{low} and IL-32^{high} melanomas from TCGA (bottom vs. top 25%, n = 118) derived by unsupervised clustering. (**C**) Percentage of lymphocyte infiltration determined from diagnostic slides accompanying melanoma samples from IL32^{low} vs. IL32^{high} (bottom and top 25%, n = 118) groups in TCGA. Two-tailed, unpaired student's *t*-test.

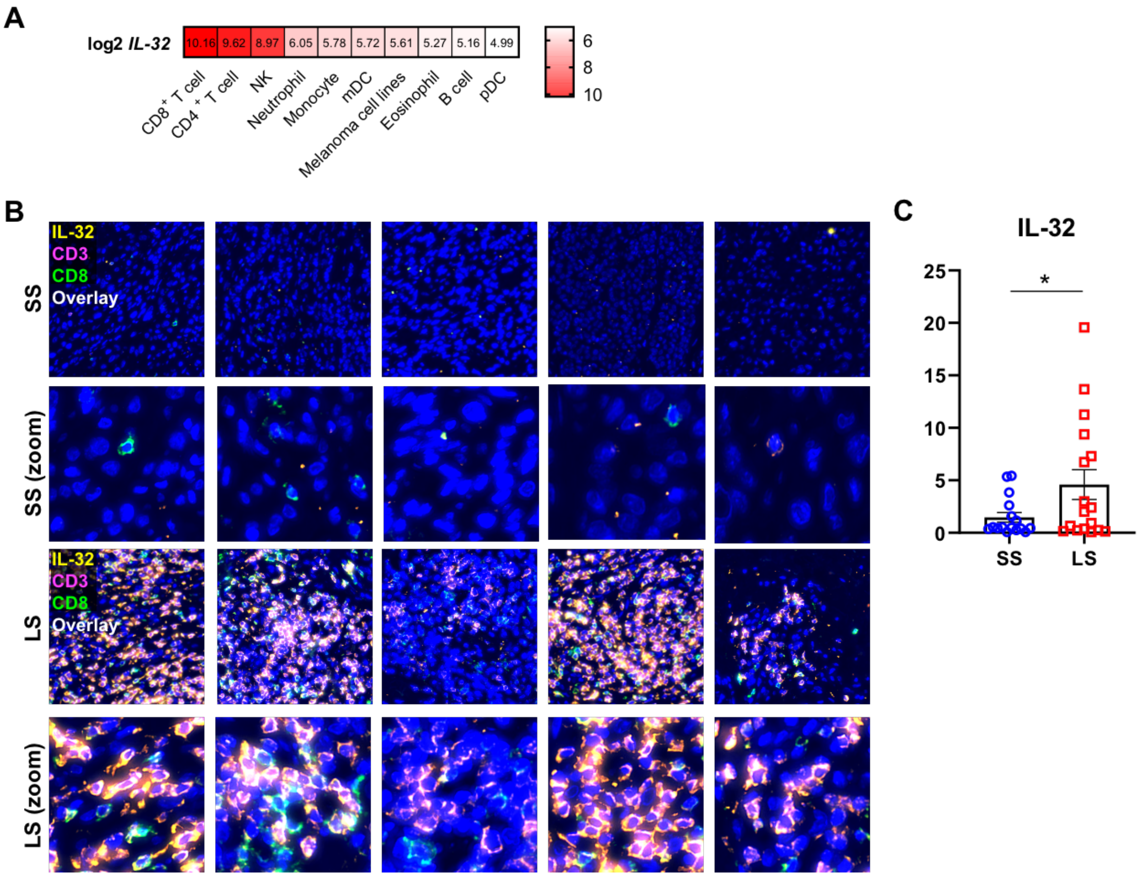

Supplemental Figure 2. IL-32 is predominantly expressed in lymphocytes. (**A**) Mean *IL32* mRNA expression in the indicated human immune cell population isolated from healthy human blood and melanoma cells (pooled from 63 melanoma cell lines). Datasets from GSE7127 and GSE28490. (**B**) Immunofluorescent images of IL-32 (yellow), CD3 (magenta) and CD8 (green) labeled FFPE melanoma tissue sections from short-term (SS < 3y) and long-term (LS > 10y) survivors and (**C**) percentage IL-32^{+} cells in melanoma tissue from short vs. long- term survivors (SS: n = 16; LS: n = 17). Two-tailed, unpaired student's *t*-test.

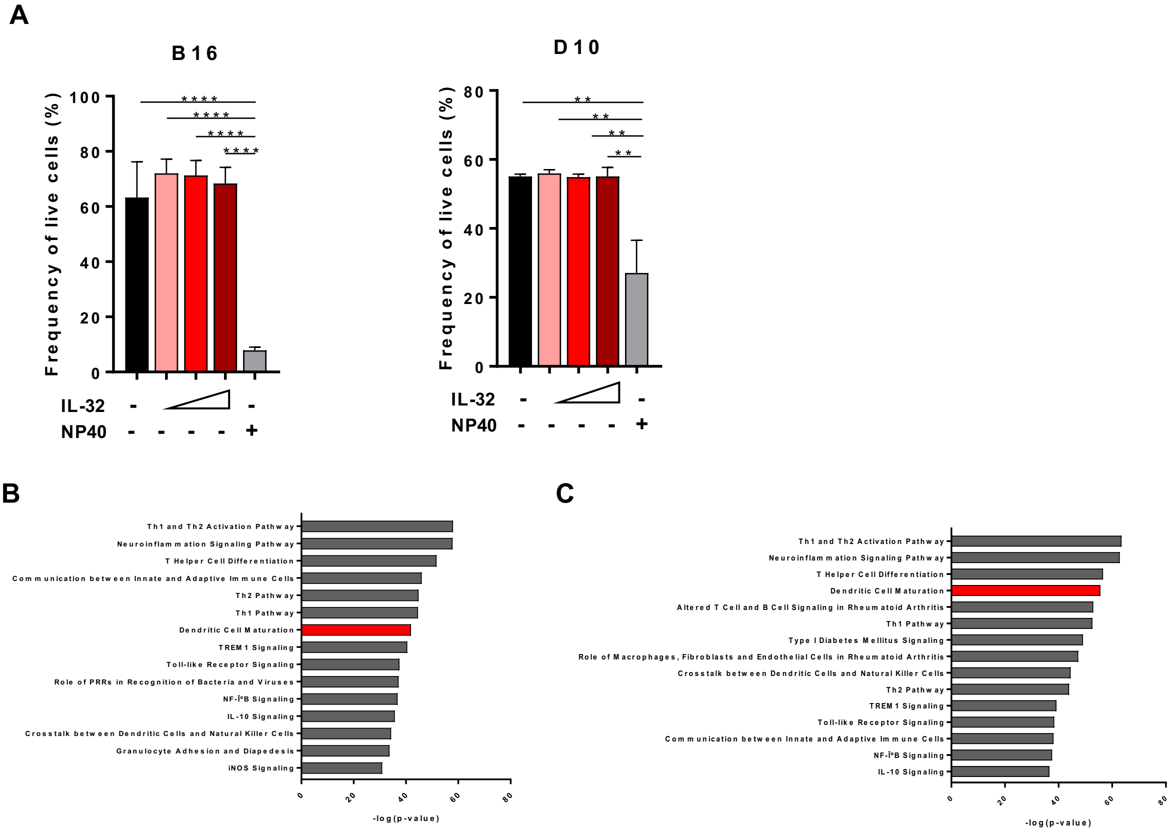

Supplemental Figure 3. IL-32 is not directly cytotoxic to B16F10 mouse melanoma and D10 human melanoma cells and induces maturation in human monocyte-derived and murine DC. (**A**) B16F10 mouse melanoma cells (n = 6) and D10 human melanoma cells (n = 3) were grown to confluence and treated with various concentrations of IL-32 (50 ng/ml, 500 ng/ml and 1 µg/ml) or NP40. After 48 hours, Annexin and PI was added to assess the frequencies of live cells by flow cytometry (Annexin$^-$/PI$^-$). Bar graphs show mean ± SEM. Statistical analysis was performed using one-way ANOVA followed by Tukey's multiple comparisons test. (**B**) Human Monocytes (n = 4) and (**C**) murine BMDC (n = 6) were treated with IL-32 or left untreated and gene expression was analyzed using Nanostring Immunology panels (human Immunology panel V2 and Mouse immunology panel, respectively). Shown are the top 15 enriched pathways as determined by Ingenuity Pathway Analysis.

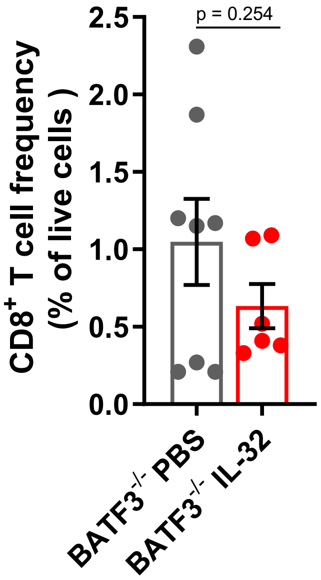

Supplemental Figure 4. IL-32 does not enhance CD8$^+$ T cell infiltration in the absence of *Batf3*-dependent DC. B16F10 tumors were established in *Batf3*$^{-/-}$ mice (n = 6-7) and treated with IL-32 or left untreated as described in **Figure 4a.** On day 12 post tumor inoculation, tumors were isolated and the frequency of CD8$^+$T cells of live cells was determined using flow cytometry. Bar graphs show mean ± SEM. Statistical analysis was performed using two-tailed, unpaired student's *t*-test.

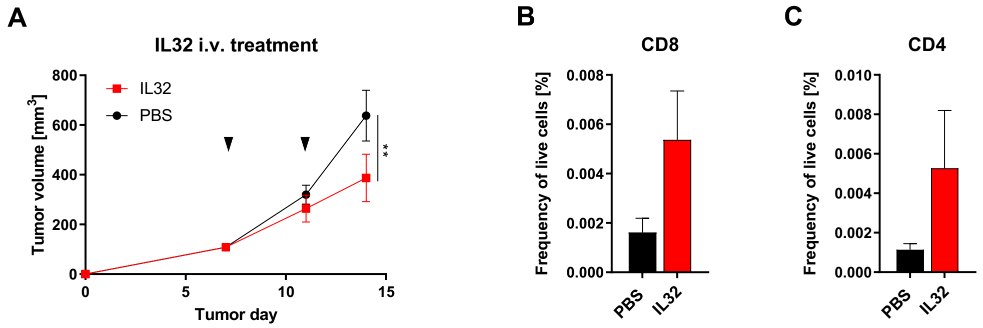

Supplemental Figure 5. Systemic IL-32 administration induces anti-tumor immunity. B16F10 tumors were established in C57BL/6J WT mice (day 0, n = 12) and treated with intravenous (i.v.) IL-32 (5ug/mouse) or PBS on day 7 and 11. (**A**) Growth curve of B16F10 melanomas in i.v. IL-32 vs. PBS treated mice, ** $p < 0.001$. On day 14 post tumor inoculation, tumors were isolated and the relative frequencies of (B) $CD8^+$ T cells and (C) $CD4^+$ T cells were determined using flow cytometry (n = 6). Data are represented as percentage of live cells. Bar graphs show mean ± SEM. Statistical analysis was performed using two-tailed, unpaired student's *t*-test.
